# Risk of missing colorectal cancer with a COVID-adapted diagnostic pathway using quantitative faecal immunochemical testing

**DOI:** 10.1093/bjsopen/zrab056

**Published:** 2021-07-06

**Authors:** Y Maeda, E Gray, J D Figueroa, P S Hall, D Weller, M G Dunlop, F V N Din

**Affiliations:** Institute of Genetics and Cancer, University of Edinburgh, Edinburgh, Scotland

## Abstract

**Background:**

COVID-19 has brought an unprecedented challenge to healthcare services. The authors’ COVID-adapted pathway for suspected bowel cancer combines two quantitative faecal immunochemical tests (qFITs) with a standard CT scan with oral preparation (CT mini-prep). The aim of this study was to estimate the degree of risk mitigation and residual risk of undiagnosed colorectal cancer.

**Method:**

Decision-tree models were developed using a combination of data from the COVID-adapted pathway (April–May 2020), a local audit of qFIT for symptomatic patients performed since 2018, relevant data (prevalence of colorectal cancer and sensitivity and specificity of diagnostic tools) obtained from literature and a local cancer data set, and expert opinion for any missing data. The considered diagnostic scenarios included: single qFIT; two qFITs; single qFIT and CT mini-prep; two qFITs and CT mini-prep (enriched pathway). These were compared to the standard diagnostic pathway (colonoscopy or CT virtual colonoscopy (CTVC)).

**Results:**

The COVID-adapted pathway included 422 patients, whereas the audit of qFIT included more than 5000 patients. The risk of missing a colorectal cancer, if present, was estimated as high as 20.2 per cent with use of a single qFIT as a triage test. Using both a second qFIT and a CT mini-prep as add-on tests reduced the risk of missed cancer to 6.49 per cent. The trade-off was an increased rate of colonoscopy or CTVC, from 287 for a single qFIT to 418 for the double qFIT and CT mini-prep combination, per 1000 patients.

**Conclusion:**

Triage using qFIT alone could lead to a high rate of missed cancers. This may be reduced using CT mini-prep as an add-on test for triage to colonoscopy or CTVC.

## Teaser

Triage of symptomatic patients referred to a colorectal service using a single quantitative faecal immunochemical test alone leads to an unacceptably high rate of missed colorectal cancers based on modelling. This may be effectively reduced by using standard CT with oral preparation as an add-on test for triage to colonoscopy or CT virtual colonoscopy.

## Introduction

COVID-19 has brought unprecedented challenges to health and social services. The standard diagnostic pathway for suspected colorectal cancer (CRC) was severely curtailed at the onset of the pandemic with the majority of hospitals missing cancer targets^1^. The screening service was halted for a period and there was a significant reduction in clinic visits, treatment initiation and follow-up across the board. Whilst vaccination programmes have started, it is clear that the pandemic will continue to restrict cancer diagnostic and treatment services due to ongoing requirements for distancing, ventilation and sanitation. Data are emerging that the COVID-19 pandemic may have lasting effects in delayed diagnosis and loss of lives and life-years^2–4^.

Many institutions have proposed alternative strategies to circumvent this situation^5–7^. One of the main approaches has been to use a quantitative faecal immunochemical test (qFIT) to stratify risk and prioritize patients for the limited number of endoscopic or radiological investigations available. Recently a few studies have advocated that this test could be used as a rule-out test for CRC, citing high negative predictive value^8^^,^^9^.

Whilst new measures are required to rationalize diagnostic resources and allocate tests to those who are most likely to have pathology, the sensitivity of the qFIT remains low compared with the established diagnostic tools in symptomatic patients. False-negative results, using qFIT as a single diagnostic test at a threshold of 10 µg haemoglobin/g faeces (µg/g), result in missed cancers^10^. Predictive value is dependent on disease prevalence and high negative predictive value is expected given the CRC prevalence is less than 50 per cent, thus it should not be treated as a marker of diagnostic accuracy^11^. Patients often do not return the test or do so with substantial delay^12^, for a variety of reasons, and thus gatekeeping patients' access to services will unnecessarily disadvantage some patients.

The Western General Hospital and University of Edinburgh have been operating a COVID-adapted diagnostic pathway, which combines multiple qFITs and a standard CT scan with oral preparation without rectal insufflation (CT mini-prep) to rule out gross colorectal pathology^13^. This iterative re-evaluation strategy could be important, not only to define the potential trade-offs but to aid clinical decision making when implementing this approach.

This paper aimed to develop models to calculate the degree of risk mitigation and estimate residual risk of CRC of the COVID-adapted pathway, considering selected alternative patterns of diagnostic pathways, and comparing them against the pre-COVID conventional diagnostic pathway.

## Methods

Decision-tree models were developed using a combination of data from the COVID-adapted pathway and a large local audit of the use of qFIT in symptomatic patients, literature review of diagnostic test accuracy and expert opinion.

The local audit of qFIT for symptomatic patients has been running since 2018 at NHS Lothian. qFIT has been used as agnostic of primary care decisionand was not utilized as a gatekeeping tool to access secondary care. There are 85 data items that have been collected in the local audit data set, including demographics, presenting history and symptoms, co-morbidities, blood results at the time of referrals and the outcome of standard diagnostic investigations (Supplementary information). In February 2021, some 5407 patients in the audit of symptomatic patients completed at least one qFIT.

The specific data derived from the COVID-adapted pathway were the sensitivity of CT mini-prep to diagnose grossly abnormal pathology, mainly CRC. CRC detection rate at the unit was calculated from a data set compiled by South East Scotland Cancer Network and the number of referrals to the department in preceding years. As most of the data about qFIT in the literature were based on asymptomatic screening populations^14^, sensitivity and specificity of a single qFIT were based on the local audit data. HM-JACKarcanalytical system (Hitachi Chemical Diagnostics Systems, Tokyo, Japan, supplied by Alpha Labs, Eastleigh, UK) based in Dundee, Scotland was used to analyse all samples. The qFIT kit was sent from a single office in secondary care. Patients returned the test kits by post to the biochemistry lab and all samples were processed in the standardized way.

The details of the COVID-adapted pathway have been published previously^15^. Briefly, patients referred by their GP under the category of ‘urgent suspicious of cancer’ (USOC) were triaged by colorectal consultants based on described symptoms and other clinical information available to:

- CT scan and qFIT if deemed 'high risk'
- Only qFIT when the referral was deemed 'low risk' or did not meet the urgent referral criteria
- Fast-track to outpatient clinic when referral documented palpable mass.

Those in the low-risk arm who had a qFIT value of 80 µg/g or above had a CT mini-prep added. Given the paucity of data in symptomatic populations at the start of the pandemic, the decision to use the threshold of 80 μg/g was pragmatic and based on the Scottish bowel-screening guidelines^16^. Considering the extreme constraints on diagnostics, it would have been counter-productive to use the cut-off of 10 μg/g as the threshold for urgent investigation as pooled data suggest the positivity rate is at least 23 per cent^10^. A second qFIT was sent to patients when the first qFIT was returned. The rationale of the two qFITs approach was based on results reported by others that it may enrich for the pathology and reduce the number of missed CRCs^17^^,^^18^.

The pathway was dynamic to the return of some diagnostics after June 2020 and changes were aimed at improving CRC detection and safeguarding; patients with qFIT values greater than 80 µg/g or equivocal CT findings underwent colonoscopy and patients with a qFIT value between 10 and 79 µg/g underwent CT colonography (CTVC); CT mini-prep was used as a safety-net measure for those who returned two qFIT values less than 10 μg/g.

The outcomes that were investigated in this evaluation were the risk of a missed CRC diagnosis and the proportion of referrals requiring a colonoscopy or CTVC. Colonoscopy and CTVC were considered equivalent for the purpose of this study. Risk of a missed CRC was considered to be a sufficient proxy measure of patient outcomes while number of colonoscopies or CTVCs are the key resource constraint in the service.

### Decision trees

Decision-tree models were developed based on plausible and potentially desirable alternative diagnostic pathways. In all cases the diagnostic pathway was modified to include triage testing (one or more qFIT, CT mini-prep). The pathway scenarios included were: option 1 – single qFIT; option 2 – two qFITs; option 3 – single qFIT and a CT mini-prep; option 4 – two qFITs and a CT mini-prep (enriched pathway). These scenarios were compared with the standard diagnostic pathway (colonoscopy or CTVC with assumed 95 per cent sensitivity at best)^19^.

Payoffs at the terminal nodes of the decision trees were limited to two binary indicators: whether or not a missed cancer occurred and whether or not a colonoscopy or CTVC was required. Node probabilities were defined based on local audit data in NHS Lothian as outlined above.

Sensitivity and specificity of colonoscopy, qFIT and CT mini-prep were obtained from the audit, SCAN data, literature and, if missing, assumptions on reasonable (best- *versus* worst-case) scenarios were made by expert opinion based on available data by the research team (3 colorectal surgeons, 2 radiologists and 1 medical statistician). As an example, CT mini-prep was used to rule out gross pathology and was not directly compared against the current standard diagnostic tests (colonoscopy or CTVC) to diagnose CRC, thus negative CT mini-prep had to be assumed truly negative in the best scenario (the actual data of 85 per cent to rule out gross pathology was assumed to be the sensitivity of detecting CRC) although the authors also considered the worst-case scenario of sensitivity of 50 per cent.

All base case parameter values are detailed in *Table 1*. Assumptions regarding symptomatic patients were required as the bulk of data regarding qFIT in the literature are based on screening populations. Since the audit of symptomatic patients has shown that the distribution and profile of qFIT values are different compared with results of symptomatic patients referred to a tertiary colorectal centre (Western General Hospital and University of Edinburgh), some estimates and assumptions were required to construct models.

**Table 1:** Base case parameter values and sources

Parameter	**Value**	Source
**Prevalence**	0.066	Audit, SCAN data
**Colonoscopy sensitivity**	0.95	Literature^12^^,^^13^
**Colonoscopy specificity**	0.99	Literature^12^^,^^13^
**qFIT 1 sensitivity (>10 µg/g)**	0.84	Audit
**qFIT 1 specificity (>10 µg/g)**	0.752	Audit
**qFIT 2 case incremental yield**	0.08	Audit
**qFIT 2 incremental TP**	0.05	Assumption
**qFIT 2 incremental FP**	0.072	Audit data and assumptions^*^
**CT mini-prep sensitivity**	0.857	COVID-adapted pathway data and expert opinion assumptions^†^
**CT mini-prep specificity**	0.914	COVID-adapted pathway data and expert opinion assumptions^†^

* The rate of patients who had first quantitative faecal immunochemical test (qFIT) negative (<80 µg/g) then second qFIT positive (>80 µg/g) has been fluctuating between 6 and 8 per cent throughout the data monitoring. However, there has been no cancer found to date within this group. Hence an assumption was made that the middle value at the point of analysis could be the false positive.

† Assumption made that truly negative standard CT scan with oral preparation (CT mini-prep) did not miss a cancer, as CT mini-prep was performed to rule out gross pathology and not directly compared with standard colonoscopy or CT virtual colonoscopy to diagnose CRC. SCAN, South East Scotland Cancer Network.

GP referrals are made in three categories: urgent suspicious of cancer (USOC), urgent and routine. To estimate the target CRC-detection rate of the COVID-adapted pathway, the prevalence of CRC from GP referrals was calculated from the number of cancers diagnosed against the number of all GP referrals received during same months in the previous years (2017–2019, excluding those referred via bowel cancer screening). The prevalence was calculated per all referrals and per USOC and urgent combined, as the latter is most likely to match the incoming GP referrals and produce the highest yield of CRC.

Incremental diagnostic yield was defined as the probability of a second qFIT value of 10 µg/g or greater when the first qFIT value was less than 10 µg/g. Incremental true positives and incremental false positives were derived from the incremental diagnostic yield under the assumption that cases and non-cases were equally likely to convert. Sensitivity and specificity of CT mini-prep were estimated from local radiologists' estimates (sensitivity 75–80 per cent) and available data in literature (sensitivity 100 per cent, specificity 87–95.7 per cent)^20^^,^^21^. However, for the base case, estimates from real-time COVID-adapted pathway data were used. As the negative CTs were not investigated further using the current standard tests (colonoscopy or CTVC) at the height of pandemic, the sensitivity and specificity of CT mini-prep from the pathway data were based on the assumption that negative CT did not miss gross CRC. CTs were reported in three categories: cancer, equivocal and normal. In the base case analysis, equivocal findings are assumed to be positive as this group is referred for the same standard tests as those for the group with a positive result. CT mini-prep sensitivity was assumed to have equivalent diagnostic accuracy in qFIT-positive and qFIT-negative populations. The sensitivity of the model to two key parameters was explored. An alternative prevalence value was considered and CT mini-prep test sensitivity was varied over a plausible range (from worst-case scenario (sensitivity of 50 per cent) to the real-time sensitivity of 85 per cent).

Decision-tree model outputs were the percentage risk of missed cancer for cancer cases, number of missed cancers per 1000 in the pathway and number of colonoscopies required per 1000 in the pathway.

The analysis was performed using software R version 4.0.0 (http://www.R-project.org) with package ‘data.tree’.

## Results

The first version of the COVID-adapted pathway was operational during the height of pandemic in April and May 2020. In the same months of preceding years (2017–2019), the average rate of CRCs detected for all the referrals (excluding bowel cancer screening but including routine referrals) was 3.8 per cent. When the referrals were restricted to a combination of USOC and urgent referrals (excluding bowel cancer screening), the average rate was 9.4 per cent.

Based on these figures, 3.8 per cent has been set as the lowest detection rate, 10.0 per cent as highest possible rate (and worst-case scenario) and 6.6 per cent (average of 3.8 and 9.4 per cent) as likely local CRC detection rate from combined USOC and urgent referrals, taking into account data from the literature (around 7 per cent)^22^^,^^23^.

Some 325 CT mini-preps were performed in the COVID-adapted pathway. Equivocal CTs were examined by two consultant radiologists. The sensitivity of detecting cancer was between 71.4 (equivocal to negative) and 85.7 per cent (equivocal to positive), assuming all normal CTs were truly normal. With these caveats in mind, the sensitivity of CT mini-prep included in the model sensitivity analysis was set between 50 (worst-case scenario) and 85.7 per cent (base case scenario).

The sensitivity of the qFIT for detecting cancer at the threshold of 10 µg/g was set as 82.5 per cent for the decision-tree model based on the authors’ local audit data of symptomatic patients over the previous year, which fluctuated between 80 and 85 per cent, in keeping with published data of qFIT sensitivity for symptomatic people^24^^,^^25^. The incremental diagnostic yield was 6–8 per cent based on the authors’ current pathway operation, which possibly offers ‘enrichment’ for pathology, that is, enhances the chance of finding a pathology.

### Base case results

**Fig. 1:**
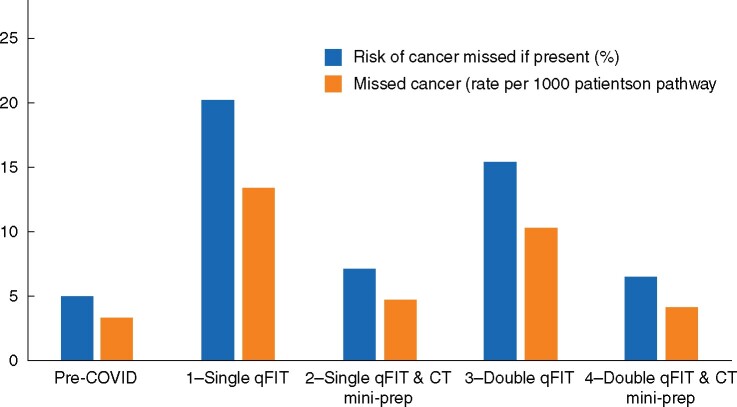
Risk of missed cancers according to diagnostic scenarios qFIT, quantitative faecal immunochemical test; CT mini-prep, standard CT scan with oral preparation

Risk of missed cancer and rate of colonoscopies for each of the alternative pathway scenarios are reported in *Table 2* and *Fig. 1*.

**Fig. 2:**
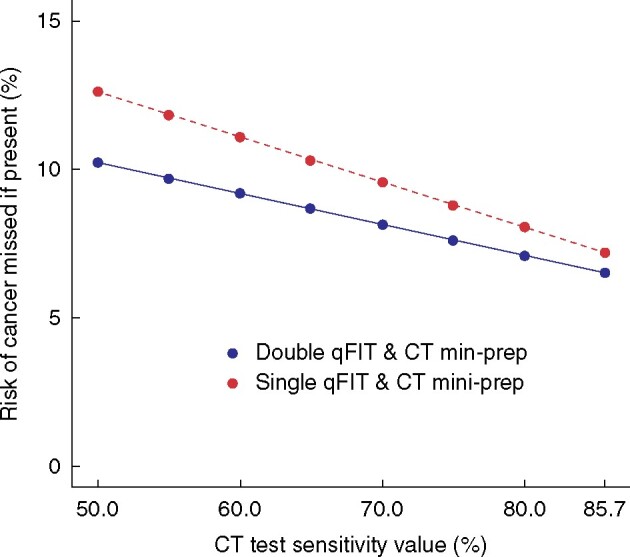
Sensitivity analysis of standard CT scan with oral preparation qFIT, quantitative faecal immunochemical test; CT mini-prep, standard CT scan with oral preparation

**Table 2:** Risk of missed cancer and colonoscopy or CT virtual colonoscopy rate, base case

Pathway scenarios	Risk of cancer being missed if present (%)	Missed cancers (rate per 1000 patients on pathway)	Colonoscopies/CTVC (rate per 1000 patients on pathway)
**Pre-COVID**	5	3.3	1000
**1 – single qFIT**	20.20	13.33	287
**2 – single qFIT plus CT mini-prep**	7.17	4.73	356
**3 – double qFIT**	15.45	10.2	358
**4 – double qFIT plus CT mini-prep**	6.49	4.29	418

CTVC, CT virtual colonoscopy; qFIT, quantitative faecal immunochemical test; CT mini-prep, standard CT scan with oral preparation.

The risk of missing a cancer, if present, was as high as 20.2 per cent with a single qFIT at the threshold of 10 µg/g used as the sole triage test. This is equivalent to approximately 13 missed cancers per 1000 patients that are evaluated using this diagnostic pathway. Using both a second qFIT and a CT mini-prep as add-on tests, the estimated risk of missed cancer reduced to 6.49 per cent, or approximately four missed cancers for every 1000 patients on the pathway. The use of CT mini-prep as an add-on test is predicted to decrease the number of missed cancers substantially compared with adding a second qFIT. The trade-off for the use of the add-on tests is an increased rate of colonoscopy or CTVC, from 287 for a single qFIT to 418 for double qFIT and CT mini-prep combination, per 1000 patients on the pathway.

### Sensitivity analysis results

*Figure* *2* shows the risk of missing cancer across a range of CT mini-prep test sensitivity values. As expected, the prevalence of CRC among the referred patients influences the rate of missed cancers and number of colonoscopies or CTVC required. Diagnostic outcomes under scenarios of 3.8 and 10 per cent prevalence are displayed in *Table 3*. Although missed CRCs are increased substantially at higher prevalence, the number of colonoscopies or CTVCs shows only a modest increase as the large majority of patients in the pathway do not have CRC.

**Table 3:** Scenario analysis, alternative prevalence values

Pathway	Missed cancers (rate per 1000 patients on pathway)		Colonoscopies/CTVC (rate per 1000 patients on pathway)	
Prevalence (%)	3.8	10	3.8	10
**Pre-COVID**	1.9	5	1000	1000
**1 – single qFIT**	7.68	20.2	270	307
**2 – single qFIT plus CT mini-prep**	2.73	7.17	338	379
**3 – double qFIT**	5.87	15.45	342	377
**4 – double qFIT plus CT mini-prep**	2.47	6.49	401	439

CTVC, CT virtual colonoscopy; qFIT, quantitative faecal immunochemical test; CT mini-prep, standard CT scan with oral preparation.

This scenario explored the impact of assuming CT mini-prep sensitivity to be as low as 50 per cent. This would result in an increase in the probability of missing a cancer to 12.61 and 10.24 per cent for options 2 and 4 respectively (8.32 and 6.75 missed cancers per 1000 patients on the pathway). This is substantially worse than the base case value although still fewer missed CRCs than under the double qFIT scenario (option 3). There would also be a slight decrease in the rate of colonoscopies or CTVCs to 335 and 400 per 1000 for options 2 and 4 respectively.

## Discussion

This COVID-adapted pathway is an example of adding a triage test sequence to an existing definitive diagnostic test^26^. The use of multiple triage tests can be thought of as ‘add-on’ testing in which a positive result at any stage leads to referral for the definitive test, while a negative result will lead to further triage testing until this pathway has been exhausted. It allows stratifying the risk to patients, to allocate limited resources optimally, particularly during the pandemic.

Various measures have been proposed to mitigate risks of missing CRCs and rationing available resources during the COVID pandemic. Understandably, there have been limited real-time data to guide whether adoption of such strategies does indeed mitigate against missing CRCs and, importantly, what residual risks are present for patients referred on such alternative pathways.

Although qFIT has been advocated by many to be a useful tool to ration limited diagnostics, caution was used in adopting the single qFIT approach due to its low sensitivity as a rule-out test in symptomatic patients. The present study shows the incremental value of adding standard CT with minimal preparation as it drives down the risk of missing CRC by 65 per cent, compared with a single qFIT.

The addition of a second qFIT does increase access to subsequent investigations, but there have been no CRCs detected in the 6.9 per cent of patients who had a positive result following an initial negative result and hence true enrichment for cancer detection cannot be calculated at this stage. Therefore, determining the value of performing double qFIT needs a longer follow-up and further data collection. Risk calculation could be further optimized by incorporating nodal information such as existing risk factors for CRC (previous advanced polyps, genetic disposition), blood results (haemoglobin, platelets) and itemized symptoms. The combination of double qFIT and CT mini-prep is substantially cheaper compared with a single colonoscopy with a similar risk of missing cancer (6.5 per cent *versus* 5 per cent for colonoscopy). Prior to the pandemic the commonest triage outcome was to offer ‘direct to test’ with minimal gatekeeping. The data presented here suggest one could further reduce the number of colonoscopies required without compromising CRC detection rate. Robust analysis of cost-effectiveness will require additional data regarding impact on health resource use in the long term, such as repeated presentation, follow-up investigations and treatment costs.

The data give transparency in terms of risks with each scenario used and the reasoning required to reach a decision about the most appropriate form of triage, given the constraints faced by the service. This information is also useful to inform GPs and patients appropriately about this alternative pathway as a reasonable option in the current resource-limited situation and give reassurance.

The major limitation of this analysis is lack of a head-to-head comparison of alternative and standard diagnostic pathways. In the absence of a randomized controlled trial or comparative study, data were retrieved from a large local audit investigating the use of qFIT for symptomatic patients with established outcomes by standard diagnostic tools. Clinical decisions must be made amidst a resource-constrained situation during the ongoing pandemic, thus the residual risks were calculated based on modelling assumptions to monitor the performance of the adapted pathway. Direct evidence could be obtained in the future from data collected in a prospective cohort study design in which patients receive all triage tests as well as the reference diagnostic test. An important caveat to the present approach, which mitigated risk by achieving similar CRC detection rates compared with previous years, was that referrals were decreased by 50 per cent, highlighting the likelihood of undetected cancers^15^.

Triage using qFIT alone leads to an unacceptably high rate of missed CRCs. This may be reduced effectively by using CT mimi-prep as an add-on test for triage to colonoscopy or CTVC. There is a need to gather more data on the diagnostic accuracy of the combined qFIT and CT pathway to validate this approach and provide confidence in the protocol.

## Supplementary Material

zrab056_Supplementary_Data
