# Supplementary material for: Risk of missing colorectal cancer with a COVID-adapted diagnostic pathway using quantitative faecal immunochemical testing
Source: BJS Open. 2021 Jul 6;5(4):zrab056. doi: 10.1093/bjsopen/zrab056 (PMC8259497; doi:10.1093/bjsopen/zrab056)
Supplement: zrab056_Supplementary_Data [file zrab056_supplementary_data.docx]

**Details of the collected data**

Community Health Index number (CHI)

Unique Hospital Patient Number (UHPI)

Age

Gender

Entry to COVID adapted pathway

Date of entry to COVID adapted pathway

Triaging consultant

Comment

Initial investigation

Initial investigation order status

Initial investigation order date

Initial investigation orer personnel

Initial send out letter category

Second investigation

Second investigation order status

Second investigation order date

Second investigation orer personnel

Second send out letter category

Third investigation

Third investigation order status

Third investigation order date

Third investigation orer personnel

Third send out letter category

End of pathway date

End of pathway letter category

End of pathway bespoke addition to letter

Letter sent by

CT appointment date

CT status

CT result

First qFIT collection date

First qFIT result date

First qFIT result

Second qFIT collection date

Second qFIT result date

Second qFIT result

Third qFIT collection date

Third qFIT result date

Third qFIT result

Presenting complaint

Abdominal mass

Rectal mass

Diarrhoea

Loose stools

Any anaemia

Iron deficiency anaemia

Rectal bleeding

Haemoglobin (Hb)

Mean cell volume (MCV)

Mean corpuscular hemoglobin concentration (MCHC)

Transferrin

Ferritin

Platelet

Past medical history

Previous history of colorectal cancer

Under surveillance for previous polyp

Known with inflammatory bowel disease (ulcerative colitis or Crohn's disease)

Currently on anticoagulant

Date added to triage or waiting list for investigation

Number of days between date of qFIT test and date added to triage or waiting list for investigation

Number of days between date of qFIT test and date of first appointment (if date of qFIT earlier)

Number of days between date of qFIT test and date of first appointment (if date of appointment earlier)

Triage priority

Initial triage outcome

Outcome/diagnosis

Normal

Cancer

Polyp

Advanced polyp

Diverticular disease

Inflammatory bowel disease

Haemorrhoid

Other lower gastrointestinal pathology

Upper gastrointestinal pathology

Second intervention/investigation (where appropriate)

Escalation of investigation basd on qFIT

Escalation/no escalation reasons

Date of second intervention/investigation

Outcome of second intervention/investigation

Comments

Pathology

Date of death

Date of data entry completion

Date of data verification date

Personnel ID
